# Assessment of the adequacy of the Fast Healthcare Interoperability Resources (FHIR) Genomics standard for the representation of somatic testing reports

**DOI:** 10.1093/jamiaopen/ooag022

**Published:** 2026-03-06

**Authors:** Robert H Dolin, Bret S E Heale, James Patterson, Kevin M Power, May Terry, Howard Anton, James Chen, Kashmira Sawant, Srikar Chamala

**Affiliations:** Elimu Informatics, El Cerrito, CA 94530-2009, United States; Humanized Health Consulting, Salt Lake City, UT, United States; MITRE Corp., McLean, VA, United States; Children’s Mercy, Kansas City, MO, United States; MITRE Corp., McLean, VA, United States; Tempus AI, Chicago, IL, United States; Tempus AI, Chicago, IL, United States; The Ohio State University, Columbus, OH, United States; Tempus AI, Chicago, IL, United States; Department of Pathology, Keck School of Medicine, University of Southern California, Los Angeles, CA 90033, United States; Department of Pathology and Laboratory Medicine, Children’s Hospital Los Angeles, Los Angeles, CA 90027, United States

**Keywords:** genomics, EHR, cancer, FHIR

## Abstract

**Objectives:**

Enabling the unambiguous communication of cancer genetic testing results and the corresponding therapeutic implications of identified genetic variants is of major clinical importance. In this report, we assess the adequacy of the Health Level Seven Fast Healthcare Interoperability Resources (FHIR) Genomics Reporting Implementation Guide, version 3 (also known as “FHIR Genomics”) for the structured communication of semantic objects typically appearing in real-world somatic testing reports.

**Materials and Methods:**

The GenomeX project team, part of the CodeX FHIR Accelerator, performed the following assessment: (1) gather a convenience sample of public somatic reports; (2) create a representative somatic report that includes a wide range of observations taken from the sample reports; and (3) structure the representative somatic report using the FHIR Genomics standard.

**Results:**

Attempted encodings were categorized into 1 of the 4 buckets: (1) full encoding possible (eg, DNA variants); (2) full encoding with potential variability (eg, therapeutic implications); (3) partial encoding (eg, genomic study details); and (4) not encoded (ie, FHIR Genomics has no explicit or acceptable semantic target, such as for coarsely granular pertinent negatives). In our sample somatic report, 47 semantic objects were noted, of which 87% had either full encoding or full encoding with potential variability.

**Conclusion:**

While dynamic real-world requirements, as exemplified by cancer genomics, often arise more rapidly than can be standardized, we conclude that the FHIR Genomics version 3 standard is sufficiently robust for the communication of current actionable somatic testing results.

## Objective

Enabling the unambiguous communication of cancer genetic testing results and the corresponding therapeutic implications of identified genetic variants has been a major focus of the Health Level Seven (HL7) Fast Healthcare Interoperability Resources (FHIR) Genomics Reporting Implementation Guide (also known as “FHIR Genomics”)^1^ since its inception in 2017.

The GenomeX project team, part of the CodeX FHIR Accelerator, sought to assess the adequacy of the latest release (version 3) of the FHIR Genomics standard for structured somatic reporting. In scope for our assessment were those semantics found in typical somatic reports, including variants, therapeutic implications, clinical trial eligibility, recommendations, specimens, testing details, and more.

## Background and significance

### Somatic reporting

Molecular profiling of tumors has become a standard part of cancer care. Its use has expanded beyond testing patients with refractory cancers to now include earlier stage patients, with the goal of providing more personalized treatment.^2–4^ As a result, precision therapies are increasingly targeted to specific stages of cancer and focus on molecular biomarkers that may be present across different cancer types. This shift toward molecularly guided therapies, shown to be more effective in specific genetic subtypes, can help avoid ineffective treatments.

Patients’ genomic testing results are commonly integrated into their electronic health record (EHR) through custom PDF reports designed by the testing laboratory. These reports include select genomic test findings, interpretation knowledge, and patient care recommendations reflecting the lab’s most current internal curation process. While state-of-the-art, these textual reports are less than ideal^5^^,^^6^—they contain only a slice of key variants and a point-in-time snapshot of interpretations; they are difficult and time-consuming to review; clinicians are not assisted in evaluating relevant interactions mentioned in the reports when making decisions; and they do not provide structured data needed for clinical decision support (CDS) guidance or analytics. To address these limitations, EHR vendors are enhancing their products in anticipation of the structured genomic findings in FHIR format and/or based on HL7 version 2 messaging.^7^ Large research projects such as eMERGE^8^ and CSER^9^ are exploring the use of FHIR Genomics, and HL7 FHIR is gaining wide traction, as are apps based on the SMART-on-FHIR platform for broader clinical applications.^10–12^

Along with the growth in testing comes a continuous growth in the discovery of tumor biomarkers known to affect prognosis or choice of therapy.^13^ As a result, there is an onus on the FHIR Genomics standard to stay current in order to provide implementation guidance for new biomarkers. Somatic testing reports may include a wide range of findings and recommendations, including variants found in a tumor specimen, germline variants with known links to somatic events such as increasing risk for developing particular cancers, molecular biomarker nongenetic observations (eg, PD-L1 or tumor mutation burden) that affect cancer care, therapeutic and prognostic implications of identified variants, oncogenicity predictions, and more.

### FHIR Genomics

The FHIR Genomics standard defines FHIR representations for a range of genomic data structures (eg, variants, haplotypes, variant implications), enabling a standards-based communication of simple and structural variants, germline and somatic variants, pharmacogenomic star alleles, HLA typing, and other findings generated from DNA sequencing, chip technology, cytogenetic analysis, along with variant annotations and interpretations. For common use cases (eg, somatic reporting, pharmacogenomics reporting, HLA reporting), FHIR Genomics provides detailed use case-specific guidance.

Maturation of FHIR Genomics is based on real-world testing and implementation feedback, including HL7 FHIR Connectathons, the HL7 FHIR “CodeX” Accelerator project, and the HL7 balloting process. HL7 FHIR Connectathons^14^ are collaborative, hands-on FHIR integration testing events held triannually. The HL7 FHIR Accelerator program^15^ is designed to assist communities and collaborative groups across the global health-care spectrum in the creation and adoption of high-quality FHIR Implementation Guides or other standard artifacts to move toward the realization of global health data interoperability. The CodeX Accelerator is focused on driving advances in cancer care.^16^^,^^17^ Under the CodeX umbrella, the GenomeX project,^18^ which is responsible for the analyses reported here, is driving advances in molecular/precision medicine (not limited to cancer). GenomeX enjoys active engagement from a number of genetic testing labs (eg, Tempus, Myriad) and EHR vendors (eg, Epic). In several cases, where the FHIR Genomics standard is not sufficiently specified for semantic interoperability, the GenomeX team asserts further constraints, via an evolving (current draft) GenomeX Data Exchange FHIR implementation guide.^19^

**Figure 1.**
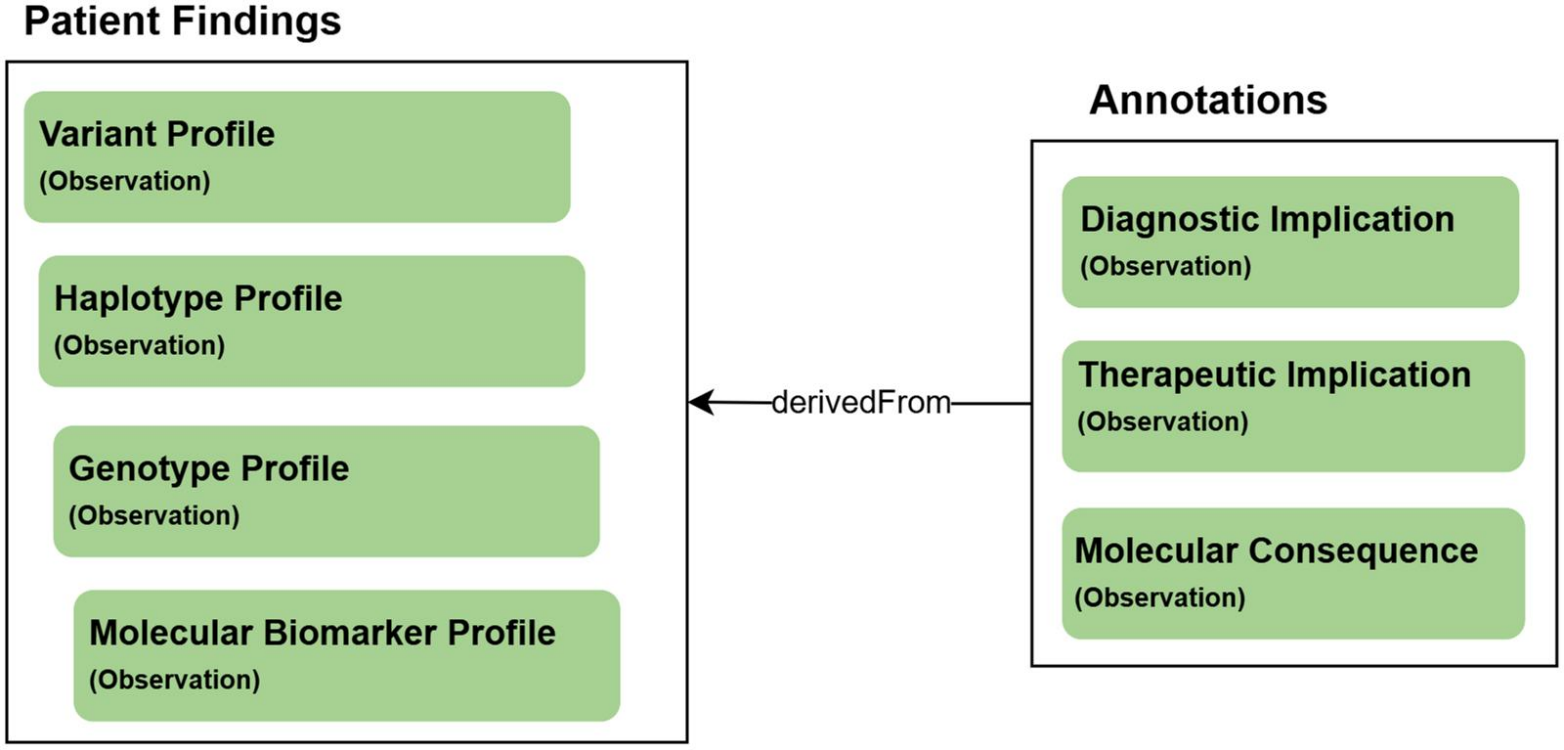
Core feature of an FHIR Genomics somatic report includes patient findings (eg, variants) and annotations derived from those findings (eg, the therapeutic implication of a variant).

At the heart of a typical FHIR Genomics somatic report are findings (variants, haplotypes, genotypes, biomarkers) and annotations derived from those findings (diagnostic implications, therapeutic implications, predicted molecular consequences), as shown in Figure 1.

**Figure 2.**
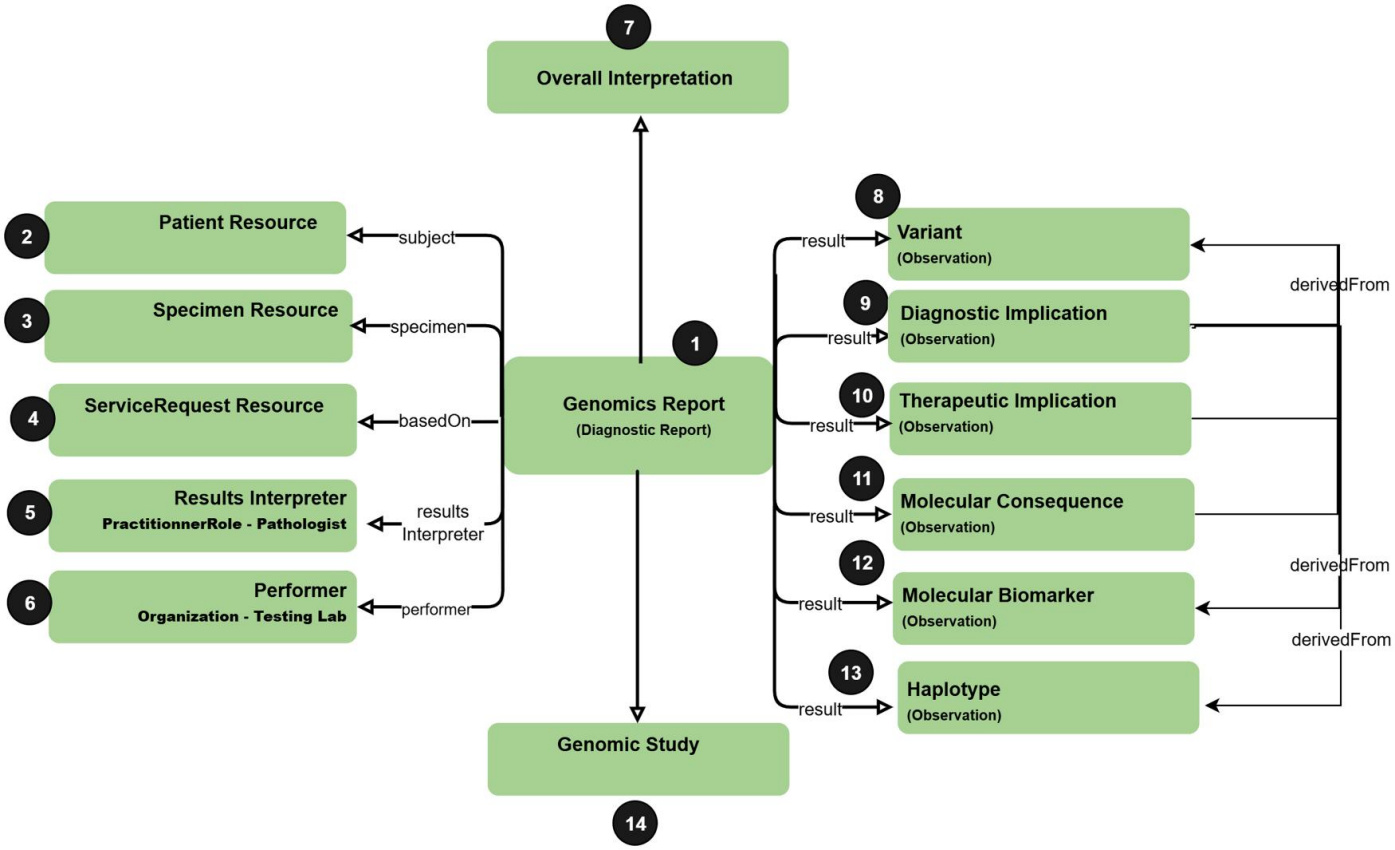
Typical arrangement of structured objects in an FHIR Genomics somatic report (circled numbers represent distinct FHIR Genomics “profiles” or “base resources,” as described in the text).

These constructs are assembled within the broader context of the Genomics Report, which generally contains information about the specimen(s), providers, and organizations involved in the ordering and testing process. The report may also contain details about the genomic study analyses used to obtain the findings, as shown in Figure 2.

Circled numbers in Figure 2 represent distinct FHIR Genomics “profiles” or “base resources,” with the former being derived from the latter. An FHIR base resource is a foundational data class (eg, “Observation,” “Patient”), which can be constrained and/or extended to create an FHIR Genomics profile (eg, the “Variant” profile is derived from the “Observation” base resource). An instance of an FHIR Genomics somatic testing report will contain data objects that conform to these base resources or profiles—for instance, a report will contain information about the patient, represented as an instance of the Patient base resource; will contain variants, represented as instances of the Variant profile of the Observation base resource; etc. FHIR tooling enables one to validate that instances conform to base resource and profile schemas defined in the FHIR Genomics standard, thereby facilitating semantic interoperability.

## Materials and methods

Several groups have examined the ability to integrate genomic data into the EHR, or, more specifically, to map genomic data reports, including cancer reports, into the FHIR Genomics standard.^9^^,^^20–23^ This prior body of work, primarily based on earlier versions of FHIR Genomics, has had an enormous impact on the evolution of the FHIR Genomics standard. Here, we extend this work by considering a wide set of real-world somatic testing reports, compared against the latest version of FHIR Genomics.

Steps in our assessment included: (1) gather a convenience sample of public somatic testing reports; (2) create a representative somatic testing report that includes a wide range of observations taken from the sample reports; (3) structure the representative somatic report using FHIR Genomics, and obvious encodings based on the FHIR base specification. The assessment was based solely on publicly available reports, and was deemed Institutional Review Board (IRB) exempt, not requiring monitoring by an IRB.

Our methods introduce a potential source of bias in that our team could have created a representative report that leaned toward inclusion of those semantic objects known to be accommodated by the current standard. To mitigate this risk, we (1) included all objects found in at least 2 reports and (2) had our senior oncologist (co-author J.C.) review the representative report for completeness.

### Gather a convenience sample

We reviewed a convenience sample of 20 somatic reports (all of which are located here^24^) Reports came from commercial and academic testing labs (eg, Caris, Foundation, Tempus, Mayo Clinic). Several of these labs are also part of the GenomeX project.

### Create a representative somatic report

While our objective did not include the development of a standard look and feel to a somatic report, we did find that most reports tended to organize different types of observations in a similar way, generally aligned with joint recommendations of the Association for Molecular Pathology (AMP), American Society of Clinical Oncology (ASCO), and College of American Pathologists (CAP).^25^ Reports generally include pertinent positives and negatives, organized with the most important (eg, actionable) findings first. Identified variants are annotated in a number of ways; all reports include indicated drugs, many reports also include molecular consequence predictions (eg, that a given variant is a frameshift mutation), variant clinical significance (or pathogenicity or oncogenicity), and potential clinical trial matches. All reports included details about the specimen(s) and testing method(s), with many reports providing details about gene coverage. Reports may include additional lab observations such as for PD-L1 biomarkers, obtained outside of sequencing. Pertinent negatives are reported at varying levels of granularity, from broad (eg, “BRAF mutations negative,” “ALK Fusions negative”) to specific (eg, BRAF V600E negative).

To construct our representative report, we crafted a PDF that adopted the general organizational structure seen in our convenience sample. We pulled in representative findings from the samples—striving to have a report reflecting a full spectrum of reported observations. Members of the HL7 Clinical Genomics Working Group and the GenomeX project reviewed and helped refine the report.

### Structure the report using FHIR Genomics

The representative report was developed as a PDF, after which we encoded the data elements in the report using the latest FHIR Genomics standard. Encoding followed the method used by the eMerge project, which entailed manually mapping report data objects into FHIR Genomics and the base FHIR specification.^23^^,^^26^ Primary FHIR encoding was performed by the co-authors and vetted with members of the HL7 Clinical Genomics Working Group and the GenomeX project. Disagreements were discussed until a singular encoding was agreed upon. We primarily strove to semantically encode content that could drive CDS. For large narrative blocks, we retained all narrative and also selectively encoded key aspects (eg, the list of genes studied).

Attempted encodings (ie, the team’s attempt to represent semantic objects in the representative report using the structured formalisms defined in the standard so as to enable semantic interoperability) were grouped into 1 of the 4 categories:

1. **Full encoding possible**: Representation in the FHIR Genomics standard is straightforward, likely to be consistent across implementations, likely to be semantically interoperable.
2. **Full encoding with potential variability**: Representation in the FHIR Genomics standard is straightforward, may have inconsistencies across implementations, likely to be semantically interoperable but requires data normalization.
3. **Partial encoding**: The FHIR Genomics standard provides guidance for only a portion of the semantic object. Inconsistencies are likely, and portions of the semantic object may be communicated as text with no additional structure.
4. **Not encoded**: The FHIR Genomics standard does not provide explicit guidance for the representation of the semantic object, and our practicing experts could not agree on a single representation. Wide inconsistencies are likely, with the inability to perform automated data normalization. Portions of the semantic object may be structured and portions of the semantic object may be communicated as text with no additional structure.

## Results

**Figure 3.**
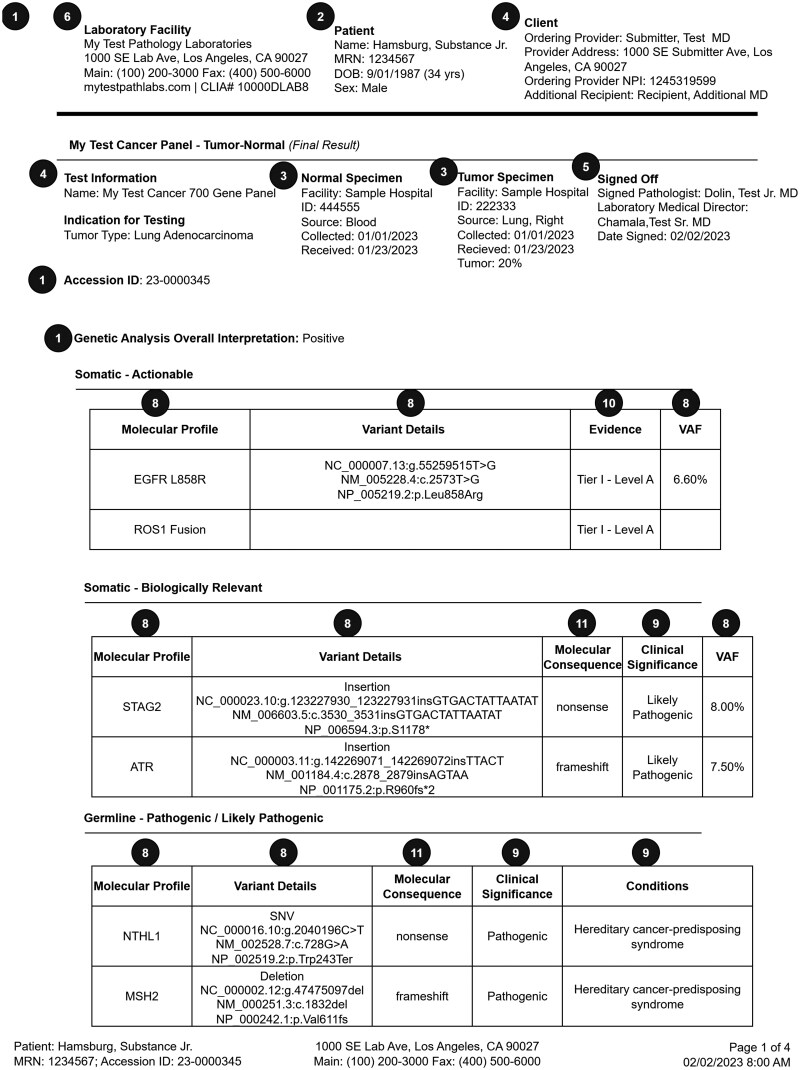
Page 1 of the final representative somatic report (circled numbers tie somatic report constructs back to the FHIR objects in Figure 2).

**Figure 4.**
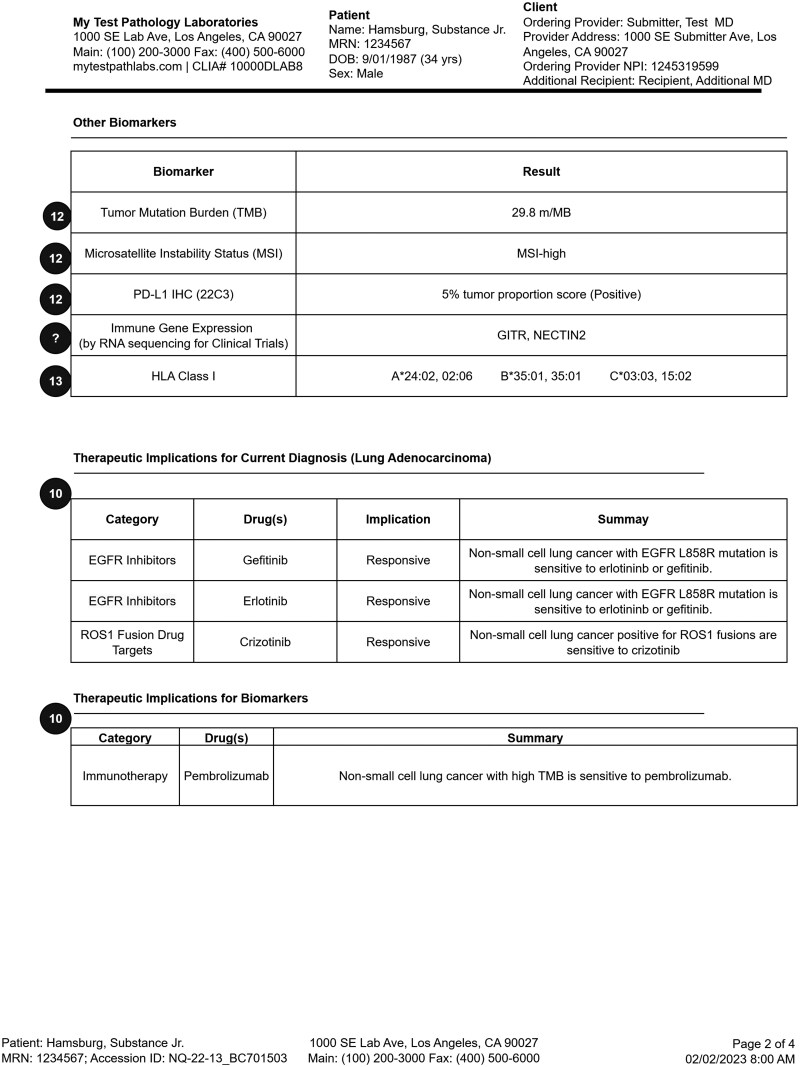
Page 2 of the final representative somatic report (circled numbers tie somatic report constructs back to the FHIR objects in Figure 2).

**Figure 5.**
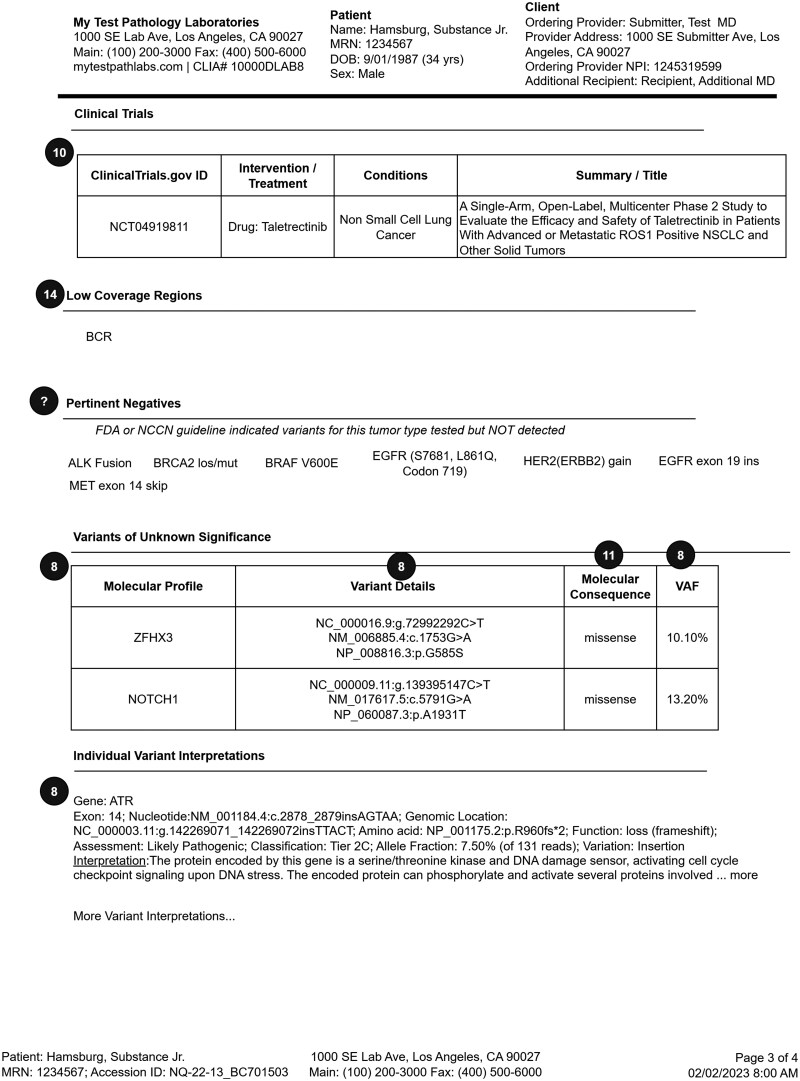
Page 3 of the final representative somatic report (circled numbers tie somatic report constructs back to the FHIR objects in Figure 2).

**Figure 6.**
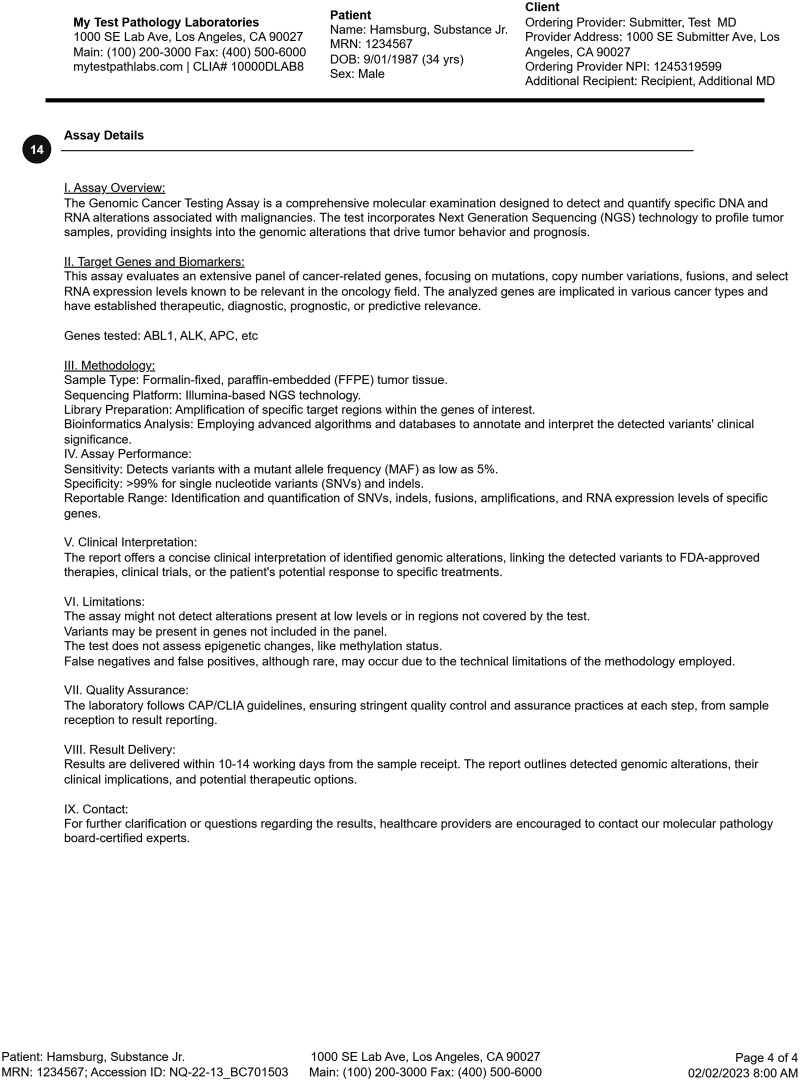
Page 4 of the final representative somatic report (circled numbers tie somatic report constructs back to the FHIR objects in Figure 2).

The final representative 4-page somatic testing report is shown in the following figures: page 1 (Figure 3), page 2 (Figure 4), page 3 (Figure 5), page 4 (Figure 6). The representative report and detailed FHIR encodings have been incorporated into the FHIR Genomics Reporting Implementation Guide as a detailed example, and are available at https://hl7.org/fhir/uv/genomics-reporting/somatics.html.

In the representative somatic testing report, the header of each page is identical, including details about the testing laboratory, basic patient demographics, and the ordering site. Page 1 (Figure 3) has information about the performed test, the specimen(s), and responsible pathologist. From there, it begins the presentation of annotated genetic findings, beginning with those most likely to be actionable. Page 2 (Figure 4) includes additional biomarker findings, where those biomarkers tested are included. Also shown on page 2 are those drugs potentially useful for this patient, based on genetic findings. Page 3 (Figure 5) includes additional clinically relevant information about potential clinical trial matches, low coverage regions, and pertinent negatives, followed by non-actionable genetic findings of lesser clinical significance. Finally, page 4 (Figure 6) includes details of the testing process, such as genes studied, the type of assay(s) used, and clinical caveats and limitations of the test.

Circled numbers in Figures 3-6 correspond to circled numbers in Figure 2, and indicate the FHIR base resource or profile to which each construct has been mapped. Circled question marks indicate those constructs for which a suitable mapping was not found in the standard. Profiles and base resources corresponding to each circled number are further described in Table 1.

**Table 1.** Description of FHIR base resources and profiles used in a somatic testing report.

#	Resource/Profile	**URL for resource/profile schema** ^a^	Description of somatic usage
1	**Genomics Report**	{baseURL}/StructureDefinition-genomic-report.html	Primary container of the overall report, including a report conclusion
2	**Patient**	http://hl7.org/fhir/R4/patient.html	The somatic report subject, typically the patient
3	**Specimen**	http://hl7.org/fhir/R4/specimen.html	The specimen(s) that this report is based on
4	**Service request**	http://hl7.org/fhir/R4/servicerequest.html	The order(s) that lead to the testing being reported
5	**Practitioner**	http://hl7.org/fhir/R4/practitioner.html	The pathologist that is responsible for the report findings
6	**Organization**	http://hl7.org/fhir/R4/organization.html	The organization that performed the reported test
8	**Variant**	{baseURL}/StructureDefinition-variant.html	The genetic variants identified via testing
9	**Diagnostic implication**	{baseURL}/StructureDefinition-diagnostic-implication.html	Phenotype implications of identified variants and other genetic findings. Generally used to communicate associated disease risk (eg, presence of a variant implies an increased risk for breast cancer). A diagnostic implication can include evidence level and clinical significance (eg, “Pathogenic”).
10	**Therapeutic implication**	{baseURL}/StructureDefinition-therapeutic-implication.html	Treatment implications of identified variants and other genetic findings. The therapeutic implication can guide or tailor the choice, dosing, or predicted effectiveness of medications or other treatments such as clinical trials. A therapeutic implication can include evidence level (eg, “tier 1—level A) and clinical significance
11	**Molecular consequences**	{baseURL}/StructureDefinition-molecular-consequence.html	The calculated or observed effect(s) of a DNA variant, generally on its downstream transcript and, if applicable, ensuing protein sequence
12	**Molecular biomarker**	{baseURL}/StructureDefinition-molecular-biomarker.html	Laboratory measurements of human inherent substances such as gene products, antigens and antibodies, and complex chemicals that result from posttranslational processing of multigene products
13	**Haplotype**	{baseURL}/StructureDefinition-haplotype.html	Generally used to convey alleles (such as HLA or pharmacogenomic star alleles) identified via testing
14	**Genomic study**	{baseURL}/StructureDefinition-genomic-study.html	The aggregated details of the specific testing, such as purpose of the test, genes tested, and type of variants tested
14	**Genomic study analysis**	{baseURL}/StructureDefinition-genomic-study-analysis.html	Each genomic study can be comprised of one or more genomic study analyses that detail the testing procedures performed

Abbreviation: FHIR, Fast Healthcare Interoperability Resources.

a Base URL=https://hl7.org/fhir/uv/genomics-reporting.

As can be seen in the figures, the vast majority of constructs in the representative report map into the FHIR Genomics standard. Circled question marks have been applied to pertinent negatives and gene expression representation. Pertinent negatives are often asserted for a category rather than for a specific finding (eg, “no ALK Fusion found,” “no BRCA2 mutation found”). While the FHIR Genomics Variant profile allows for a “present/absent” flag, the Variant profile is designed to communicate assayed variant findings and not variant categories. Therefore, while it is possible to assert something like “variant NC_000016.10: g.2040196C>T is absent,” the FHIR Genomics standard does not currently provide guidance for negating broader categories, such as “no NTHL1 variant found.” For gene expression data, while one can potentially communicate this information as a molecular biomarker, a primary challenge is that a simple comparison of absolute expression levels based on different pipelines can be misleading. Additional guidance will be needed in a future iteration of the FHIR Genomics standard to describe how to communicate normalized expression levels.

An overall semiquantitative assessment of the adequacy of the FHIR Genomics standard for the representation of somatic testing reports is shown in Table 2. The report has 47 semantic objects, of which 20 have full encoding possible, 21 have full encoding with potential variability, 3 have partial encoding, and 3 are not encoded. Taken together, 87% of semantic objects had full encoding or full encoding with potential variability.

**Table 2.** Semiquantitative assessment of the adequacy of FHIR Genomics for encoding a representative somatic testing report.

#	Mapped FHIR resource/Profile	Occurrences	Mapping category
1	**Genomics report**	1	Full encoding possible
2	**Patient**	1	Full encoding possible
3	**Specimen**	2 (normal specimen, tumor specimen)	Full encoding possible
4	**Service request**	1	Full encoding possible
5	**Practitioner**	1 (results interpreter)	Full encoding possible
6	**Organization**	1 (performer)	Full encoding possible
8	**Variant**	8 (EGFR, ROS1 Fusion, STAG2, ATR, NTHL1, MSH2, ZFHX3, NOTCH1)	7: Full encoding possible (DNA); 1: Not currently encoded (RNA)
9	**Diagnostic implication**	8 (EGFR, ROS1, STAG2, ATR, NTHL1, MSH2, ZFHX3, NOTCH1)	Full encoding with potential variability
10	**Therapeutic implication**	5 (EGFR-Drug1, EGFR-Drug2, ROS1-Drug, ROS1-ClinicalTrial, TMB-Drug)	Full encoding with potential variability
11	**Molecular consequences**	5 (ATR, NOTCH1, NTHL1, ROS1, ZFHX3)	Full encoding with potential variability
12	**Molecular biomarker**	3 (TMB, MSI, PD-L1)	Full encoding with potential variability
13	**Haplotype**	6 (HLA-A x 2, HLA-B x 2, HLA-C x 3)	Full encoding possible
14	**Genomic study**	1	Partial encoding
14	**Genomic study analysis**	2 (tumor, normal)	Partial encoding
?	**Unmapped**	2 (pertinent negatives; gene expression)	Not currently encoded

Abbreviation: FHIR, Fast Healthcare Interoperability Resources.

A summary of notable findings in the FHIR encoding of our representative somatic testing report is shown in Table 3. Note that the focus of this study was an assessment of the adequacy of FHIR Genomics, as opposed to assessing the potential for redundant representations in the standard. In other words, while we determined that FHIR Genomics provides a mechanism for the structured communication of therapeutic implication information, there remains multiple ways in which the evidence backing an asserted implication can be represented (particularly around terminology choices). Continuing maturation of FHIR Genomics profiles is based on ongoing real-world testing.

**Table 3.** Notable findings in the FHIR encoding of a representative somatic testing report.

FHIR profile	Key findings
**Diagnostic/Therapeutic implication**	**• Diagnostic and therapeutic implications** are generally straightforward to encode, with the recognition that different reports may use different evidence categorizations (eg, FDA^27^ vs AMP/ASCO/CAP^25^)
**Genomic study**	**•** Details of a **genomic study** are partially encodeable. As seen in Figure 6, details can be voluminous, and the structured components useful for CDS are evolving. A lack of international standard code sets for many aspects of a genomic study (eg, study method) lead to encoding variability
**Haplotype**	**• Haplotypes** are generally straightforward to encode, particularly for HLA genes where there is a governing body for haplotype nomenclature^28^
**Molecular biomarker**	**• Biomarkers** are generally straightforward to encode (in that they are similar to other lab observations). But many biomarkers are adopted into practice before they are assigned LOINC codes, and for many biomarkers there exists cross-lab variability in representation
**Molecular consequences**	**• Molecular consequences** are straightforward to encode, facilitated by the trend toward reporting consequences based on the MANE transcript^29^
**Variant**	**• DNA variants** are straightforward to encode **• DNA amplifications** are straightforward to encode **• RNA variants** (eg, fusions, altered splicing) do not fit neatly into the Variant profile and will likely need a new profile dedicated to RNA variants **• RNA expression levels** do not fit neatly into the Variant or Molecular Biomarker profiles and will likely need further analysis before landing on an FHIR representation **• Pertinent negatives and wildtypes** are challenging to encode given the wide range in observed granularity (eg, “NM_001354609.2: c.1799T>A” vs “EGFR exon 19 mutation” vs “BRCA2 mutation”), and the interdependence with testing scope (eg, “EGFR wildtype” generally means “to the extent that BRCA2 was studied, no variants were identified”)

Abbreviations: AMP, Association for Molecular Pathology; ASCO, American Society of Clinical Oncology; CAP, College of American Pathologists; FDA, US Food and Drug Administration; FHIR, Fast Healthcare Interoperability Resources.

## Discussion and conclusions

Overall, the FHIR Genomics version 3 standard was able to semantically structure the bulk of a representative somatic testing report. Encodings generally fell into 1 of the 4 categories:

1. **Full encoding possible**: We found that representative report content representing the patient, the performing laboratory, the specimen(s), the order(s) addressed by the current test, and the supervising pathologist could be fully encoded. We found that the Variant profile was able to fully represent the DNA variants described in the report, and that the Genomic Report profile provided a sufficient framework for capturing all aspects of the report (particularly since the Genomic Report can include narrative blocks, for those constructs not formally encoded).
2. **Full encoding with potential variability**: We found that report content representing implications (diagnostic implications, therapeutic implications, molecular consequences) and biomarkers could be fully encoded, but that without further guidance, different instances might vary in their representation. While to some extent the same could also be said about constructs in the “full encoding possible” category, the need for semantic interoperability of implications and biomarkers to drive CDS lead the team to call out the potential for redundant representations for these constructs.
3. **Partial encoding**: We found that report content representing the performed genomic study could only partially be encoded in the Genomic Study profile. This is in part a known limitation, in that the HL7 Clinical Genomics committee has made a conscious decision to focus initially on the structured representation of genomic study components likely to drive CDS, while deferring other constructs to narrative representation for now.
4. **Not currently encoded**: We found that report content representing pertinent negatives and RNA-related data have no defined semantic target in the FHIR Genomics standard, which provides no explicit guidance for these data types. These represent areas where the HL7 Clinical Genomics committee or the field could improve the coverage of the standard.

While the large majority of our semantic objects in our sample reports were fully encodable or encodable with potential variability, these observations have been added to the FHIR Genomics version 4 roadmap, and also highlight the need for ongoing FHIR Genomics updates in the highly dynamic field of cancer genomics. While real-world requirements, particularly in dynamic fields such as cancer genomics, often arise more rapidly than can be standardized, we conclude that the FHIR Genomics version 3 standard is sufficiently robust for the communication of the majority of constructs present in today’s somatic testing reports.

## Data Availability

This report summarizes an analysis that was ultimately published as part of an HL7 standard. The full FHIR Genomics standard is located at https://hl7.org/fhir/uv/genomics-reporting/STU3/ and includes the detailed somatic report located at https://hl7.org/fhir/uv/genomics-reporting/STU3/somatics.html.
